# Chlorophyll Fluorescence, Photoinhibition and Abiotic Stress: Does it Make Any Difference the Fact to Be a C3 or C4 Species?

**DOI:** 10.3389/fpls.2019.00174

**Published:** 2019-02-14

**Authors:** Lucia Guidi, Ermes Lo Piccolo, Marco Landi

**Affiliations:** ^1^Department of Agriculture, Food and Environment, University of Pisa, Pisa, Italy; ^2^Center for Climate Change Impacts, University of Pisa, Pisa, Italy

**Keywords:** environmental stress, photochemistry, photoinhibition, photosynthesis, photosystem II efficiency

## Abstract

Chlorophyll fluorescence analysis is one of the most powerful and widely used techniques to study the effect of stresses on the photosynthetic process. From the first utilization, the *F*_v_/*F*_m_ ratio has been largely used as a sensitive indicator of plant photosynthetic performance. Decreases of this index are indicative of the reduction of photosystem II (PSII) efficiency, namely photoinhibition. In the last 20 years, application of chlorophyll fluorescence has been largely improved, and many other informative parameters have been established to detect PSII photochemical efficiency and the partitioning of light energy to alternative dissipative mechanisms (qE, energy-dependent quenching; qZ, zeaxanthin-dependent quenching and qI, photoinhibitory quenching; qH, sustained photoprotective antenna quenching; qM, quenching dependent to chloroplast movement; qT, light harvesting complexes II–I state-transition) such as the recently developed “photoprotective power” of non-photochemical quenching (pNPQ). This review reports a brief description of the main chlorophyll fluorescence parameters and a wide analysis of the current bibliography on the use of different parameters which are useful to detect events of PSII photoinhibition. In addition, in view of the inherent differences in morpho-anatomical, physiological and biochemical features between C3 and C4 metabolism, possible differences in terms of photoinhibition between C3 and C4 plant species under stress conditions are proposed. The attempt is to highlight the limits of their comparison in terms of susceptibility to photoinhibition and to propose direction of future research which, assisted by chlorophyll fluorescence, should improve the knowledge of the different sensitivity of C3 and C4 to abiotic stressors.

## Photoinhibition and Stress

Photoinhibition is a phenomenon leading to a reduction of photosynthetic activity principally due to light-induced decreases in CO_2_ assimilation (Baker, 1996). Even though the reduction in photoassimilation may be dependent to damages to many components of the photosynthetic machinery, frequently the term photoinhibition is used to define light-induced inhibition of photosystem II (PSII) activity (Powles, 1984; Aro et al., 1993; Murata et al., 2007). As light is the energy needed to drive the photosynthetic process, photoinhibition is unavoidable when light exceeds the photosynthetic rate. However, the extent of photoinhibition depends on the balance between photodamage and repair mechanisms of PSII core (Demmig-Adams et al., 2012). The classical molecular scheme of photoinhibition was interpreted as the generation of reactive oxygen species (ROS) induced by excessive reduction of the primary acceptor of PSII, plastoquinone *Q*_A_, or by charge recombination between acceptor and donor side of PSII (Aro et al., 1993). The generated ROS are responsible for the damage to PSII reaction centers. However, many studies conducted on this topic give another interpretation of the photoinhibition mechanism (Murata et al., 2012): ROS do not damage PSII reaction centers directly but inhibit the repair of PSII (by inhibiting protein synthesis), with resultant stimulation of the photoinhibition of PSII. In the new scheme photodamage to PSII occurs by two consecutive steps: (i) the light-dependent destruction of the Mn cluster of the oxygen-evolving complex and (ii) the inactivation of the PSII reaction centers by light that has been absorbed by chlorophyll (Ohnishi et al., 2005).

Plants are sessile organisms subjected to daily abiotic stresses that determine detrimental effects on photosynthetic apparatus. Indeed, in addition to high sunlight, stresses such as water or mineral shortage, high and low temperature, heavy metal toxicity and air pollution, can determine at what point the light absorbed by chlorophyll pigments becomes excessive for the requirement of photosynthetic machinery (Murata et al., 2007). The first hypothesis on the effects of environmental stresses on PSII activity suggested that stressors accelerate the photoinhibition to PSII (Björkman and Powles, 1984; Melis, 1999; Adir et al., 2003). Recently, this hypothesis has changed and many researchers have demonstrated that the repair mechanism of PSII is more sensitive to environmental stresses than the process of photodamage itself (Nishiyama et al., 2001; Allakhverdiev and Murata, 2004; Takahashi and Murata, 2008; Kangasjärvi et al., 2012; Nishiyama and Murata, 2014). Photosystem II is the most susceptible component to be damaged in the thylakoid membranes. Therefore the principal result of abiotic stress is to make PSII prone to photoinhibition (Nishiyama et al., 2006). On the contrary, PSI is less frequently damaged due to a very efficient photoprotection mechanism which can prevent photoinhibition (Gururani et al., 2015). Photoinhibition to PSI occurs when the supply of electrons from PSII exceeds its capacity to accept electrons (Tikkanen and Grebe, 2018). The recovery process in PSI, when photodamaged, is prolonged (Tikkanen et al., 2014) and in some cases not wholly reversible (Kudoh and Sonoike, 2002). However, the protective mechanisms involved in PSI photoinhibition are not well-understood, because studied PSI photoinhibition occurred in isolated thylakoids under non-stressed conditions, or *in vivo* only in specific environmental conditions and species-specific manner (Teicher et al., 2000; Zhang and Scheller, 2004; Sonoike, 2011; Zivcak et al., 2015). Nonetheless, some studies revealed that some factors regulate PSI photoprotection, i.e., the proton gradient dependent or *cyt* b6f-mediated slow-down of electron transport rate (Joliot and Johnson, 2011; Suorsa et al., 2012) and the light-harvesting of PSII (LHCII)-mediated excitation of PSII and PSI via non-photochemical quenching and the phosphorylation of LCHII (Grieco et al., 2012). In addition to these factors, Tikkanen et al. (2014) reported that PSII photoinhibition slows down the electron transport rate and prevents ROS generation and photodamage to PSI. Moreover, Ballottari et al. (2014) reported a zeaxanthin-dependent regulation of PSI functional antenna size in *Arabidopsis thaliana*. The authors showed that similar to its action in PSII, zeaxanthin binding to components of PSI leads to the formation of carotenoid radical cations, quenching a portion of the excitation energy absorbed.

The phenomenon of photoinhibition has been studied for well over a century by using multiple biochemical, biophysical and genetic approaches. Probably, chlorophyll fluorescence is one of the most utilized ecophysiological techniques to study the photosynthetic process in plants (Murchie and Lawson, 2013) as evidenced by the high number of user-friendly, non-invasive and portable chlorophyll fluorometers that are available today. However, despite the simplicity of the utilization of chlorophyll fluorometers, the theory and interpretation of data arising from chlorophyll fluorescence measurements is still a complex matter. Many reviews report the theoretical background of chlorophyll fluorescence analysis and a huge body of research reports the utilization of this methodology to provide information related to the photosynthetic process (Maxwell and Johnson, 2000; Baker, 2008; Guidi and Calatayud, 2014; Kalaji et al., 2014, 2017; Guo and Tan, 2015; Ruban, 2016; Stirbet et al., 2018). The first important parameter derived from the Kautsky curve was the *F*_v_*/F*_m_ ratio (Krause, 1988) and subsequently, it became a key parameter to detect the PSII photoinhibition induced by a stress factor (Krause and Weis, 1991). To determine this ratio, a weak modulated measuring beam is applied to determine minimal fluorescence yield in a dark-adapted leaf (*F*_0_), and a saturating flash is then superimposed to induce the maximal yield of chlorophyll fluorescence (*F*_m_). The ratio *F*_v_/*F*_m_ [(*F*_m_ -*F*_0_)/*F*_m_] represents an estimator of the maximal photochemical efficiency of PSII and is utilized to detect the loss of function of PSII reaction centers (Öquist et al., 1992). Values of *F*_v_/*F*_m_ ranges typically between 0.75 and 0.85, and this ratio is proportional to the quantum yield of photochemistry (Kitajima and Butler, 1975). A decline of this ratio is considered to be a good indicator of photoinhibition that may result from two different processes (Öquist et al., 1992): a decrease in the rate constant of PSII photochemistry caused by damages to the PSII reaction centers and/or an increase in the rate constant of non-radiative dissipation of excitation energy. The decrease in the rate constant for PSII photochemistry leads to a rise in initial fluorescence at open PSII traps (*F*_0_) whereas an increase in the rate constant of non-radiative energy dissipation leads to a decrease in both initial fluorescence (*F*_0_), and maximum fluorescence at closed PSII traps (*F*_m_) (Kitajima and Butler, 1975). However, sometimes the decrease of *F*_v_/*F*_m_ ratio is not linearly related to the amount of inactivated PSII reaction centers (Park et al., 1996). Indeed, it is worthwhile to underline that the *F*_v_/*F*_m_ ratio is sometimes considered (erroneously) as an indicator of PSII photoinactivation, but this ratio decreases not only due to the closure of PSII reaction centers but also when other processes compete with charge separation such as the thermal dissipation of absorbed light (Malnoë, 2018), as detailed below.

Although the parameters reported above can provide useful information, the introduction of chlorophyll fluorescence quenching analysis has given a further advance in the detection of PSII photoinhibition (van Kooten and Snel, 1990; Bolhàr-Nordenkampf and Öquist, 1993). Quenching analysis permits the separation of the contributions of photochemical and non-photochemical processes in the quenching of variable fluorescence, by inducing a temporary closure of all PSII reaction centers by a strong saturating light pulse (Schreiber et al., 1995; Baker, 2008). The decrease in fluorescence due to photochemistry, i.e., the charge separation, is named photochemical quenching. Regarding photochemistry, the most useful parameter derived from quenching analysis is the measure of the efficiency of PSII (Φ_PSII_; Genty et al., 1989). Superficially similar to Φ_PSII_, another parameter which may be derived from quenching analysis is the coefficient of photochemical quenching, qP, that indicates the proportion of open PSII reaction centers (Maxwell and Johnson, 2000). The parameter Φ_PSII_ provides information on the electron transport rate and, differently to the *F*_v_/*F*_m_ ratio (determined in dark-adapted conditions) on the nature of photoinhibition. Indeed, the decline in Φ_PSII_ is due to the inactivation of PSII reaction centers aimed at photoprotection (Krause et al., 1990) or may be a mechanism which adjusts the efficiency of PSII to photosynthetic photon flux density (Critchley, 1994). Besides, under light Φ_PSII_ depends on the activity of energy-consuming biochemical reactions of CO_2_ assimilation (Genty et al., 1989). Besides the proportion of light energy tunneled to photochemistry, quenching analysis can be used to determine the amount of light energy dissipated by alternative mechanisms, namely non-photochemical quenching (Logan et al., 2014). With quenching analysis, it is indeed possible to identify a key parameter, the non-photochemical quenching (NPQ), that represents the fastest process of rapid and reversible thermal dissipation of absorbed light energy in the PSII antenna (Niyogi, 2000; Müller et al., 2001; Horton and Ruban, 2005; Ruban et al., 2012).

Even though NPQ is viewed as a dissipation mechanism into heat, several components are involved: the energy-dependent (qE), zeaxanthin-dependent (qZ) and photoinhibitory quenching (qI) (Derks et al., 2015). Among these mechanisms, qE and qZ are essential for photoprotection while qI could represent the photoinhibitory damage to PSII reaction centers (Ruban et al., 2012). qE is the fastest and most effective component at photoprotecting PSII reaction centers from damage (Nilkens et al., 2010; Goss and Lepetit, 2014). In excess light conditions, a transthylakoidal proton gradient (ΔpH) is generated, activating qE (Noctor et al., 1993). Acidification of thylakoid lumen induces the protonation of the PSII subunit S (PSbS) protein, which then activates violaxanthin epo-oxidase, which in turn converts violaxanthin (Vio) into zeaxanthin (Zea) (Demmig-Adams, 1990). Both PsbS and Zea act as allosteric modulators that increase the sensitivity of LHCII to the lumen protons inducing qE (Horton et al., 2000; Ruban et al., 2012). The coefficient qZ was characterized by Dall’Osto et al. (2005) and Nilkens et al. (2010); it is formed within 10–30 min, and it is independent to PSbS and ΔpH even though strictly dependent to Zea epoxidation and induces conformational changes of the minor antenna protein CP26. qE and qZ, determine changes of the LHCII, while the last quenching coefficient qI is relatively slower to relax (hours or more), and involves a loss in the number of active PSII reaction centers from photoinhibition (Derks et al., 2015). The qI coefficient is the result of photoinhibition (Baker, 1996) and is due predominantly to inactivation and/or degradation of D1 protein. Nevertheless, qI depends only partially to D1 degradation (Demmig and Björkman, 1987; Chow et al., 1989) and this independent portion has been recently named qH (Malnoë et al., 2017) that encompasses different processes. Some of which aimed at photoprotecting, occur in the antenna possibly with similar mechanisms involved in qE and qZ. However, these components act in a different way to dissipate the excess of excitation energy and are involved in the adaptive response to environmental constraints (Malnoë et al., 2017). The qH coefficient gives information on the decrease of the *F*_v_/*F*_m_ ratio. Indeed, a decrease in the ratio can be attributable to a high *F*_0_ (due to the inactivation of PSI reaction centers or to the antenna detachment), but also to both decrease in *F*_0_ and *F*_m_ attributable to the presence of qH.

In theory, the components of NPQ can be separated, but practically this not always a simple matter due to the heterogeneity of their kinetics of induction and relaxation that can provide sometimes misleading results, i.e., the overlapping of the recovery time of qZ and qI (Nilkens et al., 2010). Therefore, as both photoinhibition/photodamage and NPQ compromise the measure of Φ_PSII_ (Ruban and Murchie, 2012), a new fluorescence methodology has been developed to assay *in vivo* the protective potential of NPQ (Ruban and Murchie, 2012; Ruban and Belgio, 2014). The method consists in monitoring photoinhibition that results from the decline in qP in the dark, measured immediately after illumination to determine the parameter qPd. Both qPd and NPQ parameters are utilized to fit Φ_PSII_. The methodology allows the separation of the effectiveness of photoprotective NPQ from the photoinhibitory effect occurring in leaves (Ruban and Belgio, 2014; Giovagnetti and Ruban, 2015; Ware et al., 2015; Lo Piccolo et al., 2018).

Usually, the term NPQ is related to the dissipation of excess light energy absorbed as heat, even though some mechanisms quench the excess of excitation energy without heat dissipation. Among processes that do not dissipate excess energy as heat (Malnoë, 2018), fluorescence decline can be dependent to chloroplast movement induced by white or blue actinic light (the component qM; Cazzaniga et al., 2013; Dall’Osto et al., 2014). Even the re-distribution of energy between the two photosystems determines a decrease in chlorophyll fluorescence at room temperature: the process results in the II–I state transition and is due to the movement of phosphorylated LHCII away from PSII (Quick and Stitt, 1989). This component of NPQ is termed qT even though it does not contribute significantly to NPQ at saturating light intensities (Nilkens et al., 2010) and it can also be depressed under high light conditions (Mekala et al., 2015). Ferroni et al. (2014) reported in lycophytes an extra-qT mechanism that could reflect the use of PSI as an energy quencher for PSII through an energy spillover mechanism. Interestingly, energy spillover to PSI was also suggested to be functionally relevant in angiosperms, in particular when the light intensity becomes so high that the plant’s capacity for qE is saturated, and is therefore insufficient for effective photoprotection (Ferroni et al., 2016; Tiwari et al., 2016).

In conclusion, PSII photoinhibition represents a mechanism by which plants limit the photodamage to PSII but also preserves PSI which is not equipped with its own repair mechanisms (Sonoike, 2011; Tikkanen et al., 2014; Järvi et al., 2015). In conclusion, photoinhibition of PSII was previously considered as a solely negative mechanism to limit photodamage to PSII, effectively limiting the photosynthetic process. The new perspective of PSII photoinhibition also understands it as a means to protect PSI, which is not equipped with its own repair mechanisms.

## Photoinhibition in C3 and C4 Species

It is generally accepted that C4 plants such as maize, sorghum, and sugarcane have impressively higher levels of photosynthetic efficiency than most C3 species, such as wheat and rice (Kajala et al., 2011). This is attributable to the different mechanism of carbon fixation connected to both morpho-anatomical and biochemical differences existing between C3 and C4 species. C3 photosynthesis only uses the Calvin cycle to fix CO_2_, an event which is catalyzed by ribulose-1,5-bisphosphate carboxylase (Rubisco) and takes place inside of the chloroplasts of mesophyll cells (MC). Conversely, in C4 species the photosynthetic activities are partitioned between mesophyll and bundle sheath cells (BSC) that are anatomically and biochemically distinct. In C4 photosynthesis, the first step of carbon fixation is catalyzed by phosphoenolpyruvate (PEP) carboxylase which conjugates CO_2_ to PEP forming oxaloacetate (OAA). Then, OAA is reduced to malate, which then diffuses into the BSC where it is decarboxylated, and CO_2_ enters the Calvin cycle. In this way, the C4 photosynthetic metabolism operates a CO_2_-concentrating mechanism around Rubisco, which in turn significantly reduces the Rubisco oxygenase activity. In addition, BSC chloroplasts contain low level of PSII (Romanowska et al., 2017) and, although in BSC chloroplasts PSII contains all polypeptides involved in electron transport and oxygen evolution, it is largely inactive (Romanowska et al., 2008). In contrast, MC chloroplasts have higher PSII activity but practically absent Rubisco activity (Furbank and Foyer, 1988).

The metabolic mechanism in C4 species increases the energy utilization efficiency by reducing the subsequent energy loss due to photorespiration, as occurs in C3 species, which results in higher photosynthetic performances and water use efficiency of C4 when compared to C3 metabolism (Majeran et al., 2010). This CO_2_ concentrating mechanism in C4 metabolism is thought to have evolved in response to declining atmospheric CO_2_ concentrations over geological time scales (Ehleringer et al., 1991). A higher water use efficiency is attributable to the fact that the CO_2_ concentrating mechanism allows C4 species to maintain a large diffusion gradient for CO_2_ and operate at lower leaf conductance than C3 species, thereby reducing water loss by transpiration (Long, 1999). Besides these general features, C4 species are further classified into subtypes according to decarboxylation reaction that they utilized, a feature which makes an additional difference in their energy use efficiency and, in turn, their photosynthetic yield: NADP-malic enzyme (NADP-ME), NAD-malic enzyme (NAD-ME) and PEP carboxykinase (PEPCK) (Hatch, 1987). However, no “pure” PEPCK-type C4 species have been actually described, and currently NAD-ME (e.g., *Panicum virgatum, Pennisetum glaucum, Amaranthus* spp.) and NADP-ME subtypes (e.g., *Zea mays, Saccharum* spp., and *Sorghum bicolor*) are suggested as distinct C4 biochemical pathways, both with or without the additional service of the PEPCK pathway (Rao and Dixon, 2016). All these features make C4 species better adapted to high light intensities and high temperatures, and therefore C4 species are mainly present in warmer areas, such as the tropical/subtropical regions (Edwards et al., 2010). Given that different abiotic stressors (e.g., drought, salinity, and cold) lead in most cases to a surplus of energy absorbed by the leaves in relation to a stress-altered CO_2_ assimilation, we might expect C4 species to be less prone to photoinhibition and photodamage than C3 under abiotic stress, especially under limited water availability (i.e., drought and salinity). But is this the truth? And connected to this, why do C4 species curiously only represent less than 4% of the total world’s flora (Ghannoum, 2009)? And “why are there no C4 forests?” (Sage and Sultmanis, 2016).

Unfortunately, there is little information concerning the comparison between C3 and C4 photosynthetic performances under the same stressful conditions, and even less information is available on the susceptibility of photoinhibition in C4 versus C3 species subjected to abiotic stressors. Besides the difficulty of drawing a clear picture between C4 and C3 photosynthesis under stress which arises from differences of experimental conditions, the situation is further complicated by the different C4 metabolisms (NADP-ME, NAD-ME, and PEPCK). Below, on the basis of the inherent differences between C3 and C4 photosynthesis, and revising the few reports on the subject, we attempt to solve the question as to whether C4 species are or are not less prone to photoinhibition when exposed to abiotic stresses. We are aware that (i) different results about the inherently lower or similar values of *F*_v_/*F*_m_ ratio in C4 than C3 species (Table 1) and (ii) different experimental conditions, sometimes more favorable to C3 or C4 species, further complicate the comparison and (iii) MC present greater photodamage than BSC (Pokorska et al., 2009).

**Table 1:** Values of the *F*_v_/*F*_m_ ratio in C3, C3-C4, and C4 species determined in different experimental conditions.

Species		*F*_v_/*F*_m_ ratio	Experimental conditions	Reference
*Spartina maritima* (Curtis) Fernand and *S. densiflora* Brong *Arthrocnemum perenne* (Miller) Moss and *A. fruticosum* (L). Moq	C4 C3	0.74–0.72 0.65–0.71	Midday condition: 31 ± 0.3°C, photosynthetic flux density (PFD) of 1370 ± 10 μmol m^-2^s^-1^ (12/12, light/dark) 41 ± 1% RH	Nieva et al., 1999
*Cleome spinosa* L. *C. gynandra* L.	C3 C4	0.859 ± 0.001 0.805 ± 0.005	Seedlings were grown for 3 weeks under controlled conditions (light/dark regime of 16/8 h at 25°C, RH of 70%, PFD of 350 μmol m^-2^ s^-1^)	Uzilday et al., 2012
*Zea mays* L. ‘Golden Bantam’	C4	0.80	The growth room was controlled at 30/25°C (light/dark), relative humidity of about 70%, a 12-h photoperiod (6:00–18:00) and light intensity of 600 μm m^-2^ s^-1^	Hasan et al., 2006
*Sorghum bicolor* L. ‘Liaoza 10’	C4	0.80	Water culture in a greenhouse with a maximum irradiance of 1217 ± 26 mmol m^-2^ s^-1^ and a day/night temperature of 35/22°C. RH was 40–60%.	Jiang et al., 2011
*Eragrostis minor* Host	C4	0.79 ± 0.006	Maintained under a 14 h photoperiod with a PFD of 600 μmol m^-2^ s^-1^ measured at plant height, day/night temperature of 25/15°C, and RH of 60/80% for 12 weeks	Liu and Osborne, 2008
*Z. mays* hybrid Zhengdan958 *Nicotiana tabacum* K236	C4 C3	0.771 0.832	Maintained in pots at a PFD of 1000 μmol m^-2^ s^-1^ with 14/10 h of light/dark cycle at 24/22°C (day/night)	Ruan et al., 2017
*Muhlenbergia glomerata* (Willd.) Trin. *Calamagrostis canadensis* (Michx.) Beauv.	C4 C3	0.77 ± 0.01 0.77 ± 0.01	Maintained In pots at a PFD of 800 μmol m^-2^ s^-1^ with 14/10 h of light/dark cycle at 26/22°C (day/night) and 70/80% RH	Kubien and Sage, 2004
*Miscanthus x giganteus* (Greef & Deuter ex Hodkinson & Renvoize) *Z. mays* genotype FR1064	C4 C4	0.76 ± 0.01 0.79 ± 0.01	Maintained In pots at a PFD of 500 μmol m^-2^ s^-1^ at 25/20°C (day/night) and 70% RH	Naidu and Long, 2004
*Z. mays Digitaria sanguinalis* (L.) Scop. *Echinochloa crus-galli* (L.) Beauv.	C4 C4 C4	0.79 ± 0.004 0.80 ± 0.010 0.81 ± 0.007	Grown in hydroponic at a PFD of 200 μmol m^-2^ s^-1^ at 14 h of photoperiod, 24/21°C (day/night)	Romanowska et al., 2017
*Z. mays* cultivar Ardiles Banguy Fjord Magister	C4	0.808 0.808 0.803 0.791	Maintained In pots at a PFD of 500 μmol m^-2^ s^-1^ at 20°C	Lootens et al., 2004
*Panicum coloratum* L. *Cenchrus ciliaris* L. *Flaveria bidentis* L. (Kuntze)	C4 C4 C4	0.779 ± 0.004 0.790 ± 0.002 0.790 ± 0.002	Maintained at a PFD of 550 μmol m^-2^ s^-1^ at 10 h of photoperiod, 25/20°C (day/night) and 70% RH	Dwyer et al., 2007
*Flaveria cronquistii* A.M. Powell *F. cronquistii x brownii* *F. pringlei* Gand. *F. brownii x F. cronquistii* *F. linearis* Lag. *F. chloraefolia* A. Gray *F. anomala* B.L. Rob *F. pubescens* Rydb. *F. floridana* J.R. Johnst *F. brownii* A.M.Powell *F. australica* Hook *F. bidentis* *F. palmeri* J.R. Johnst *F. trinervia* (Spreng.) C.Mohr *Z. mays*	C3 C3 C3 C3 C3–C4 C3–C4 C3–C4 C3–C4 C3–C4 C4-like C4 C4 C4-like C4 C4	0.840 ± 0.008 0.837 ± 0.001 0.833 ± 0.006 0.835 ± 0.006 0.820 ± 0.008 0.818 ± 0.015 0.815 ± 0.019 0.813 ± 0.006 0.826 ± 0.007 0.805 ± 0.006 0.768 ± 0.002 0.768 ± 0.002 0.773 ± 0.006 0.767 ± 0.005 0.789 ± 0.007	Not reported	Pfündel, 1998
*Arabidopsis thaliana* (L.) Heynh. *Z. mays*	C3 C4	0.84 ± 0.003 0.80 ± 0.012	Plants were grown on vermiculite in a growth chamber under a 14-h photoperiod and a day/night regime of 24/22°C, at an irradiance of 200 μmol photons m^-2^ s^-1^.	Zienkiewicz et al., 2015


As explained above, water use efficiency is typically higher in C4 than C3 species, and it is linked to the CO_2_ concentrating mechanisms in C4 photosynthetic metabolism (Downes, 1969). However, stomatal limitation posed by a stressor (e.g., drought) potentially induces a reduction in photosynthetic CO_2_ uptake at a large extent in C4 species than in C3 species, because C4 photosynthesis operates at (or close to) the inflection point of the photosynthetic CO_2_ response (Wand et al., 2001). Under drought-controlled conditions, Ripley et al. (2007) demonstrated that, besides stomatal limitations, a lower CO_2_ assimilation rate in C4 versus C3 subspecies of *Alloteropsis semialata* was caused by a lower linear electron flux and a reduction of PSII photochemical efficiency under water limitation, rather than in differences between increased electron flux toward alternative sinks. Similarly, Killi et al. (2017) found that different maize genotypes had a higher decline of *F*_v_/*F*_m_ and Φ_PSII_ than sunflower genotypes under water stress conditions. The authors also observed that the combination of drought and high temperature (35°C) resulted again in higher photoinhibition and a lower PSII efficiency in maize than sunflower, thereby suggesting that C4 species are poorer performers than C3 species under stressful conditions, even at a temperature for which the C3 metabolism should be disadvantaged. In other cases, it has been observed that the limited capacity for photorespiration or the Mehler reaction (which act as significant alternative electron sinks in C3 species) was deleterious under water stress, a condition in which the absorbed light largely exceeded the carboxylation energy requirement (Ghannoum, 2009). Practically, this may explain why C4 is equally or in most cases even more sensitive to water stress than C3 photosynthesis (despite the greater water use efficiency of the C4 species), and clarify the paradox of why C4 species abundance declines in parallel with decreasing in annual rainfall (Paruelo and Lauenroth, 1996; Tieszen et al., 1997).

Another well-known factor that increases the probability of photoinhibition in plants is the exposure to low temperatures, especially when occurring in conjunction with high light (Pietrini and Massacci, 1998), but possible differences in C3 versus C4 species are less obvious. C4 species typically have a reduced amount of Rubisco in BSC chloroplasts as compared to C3 MC, and therefore BSC reactions can represent a key limiting factor at low temperature, a condition in which the CO_2_ solubility, and consequently, its diffusion is reduced (Kubien et al., 2003). Furthermore, C3 plants have a higher maximum quantum yield of CO_2_ than C4 plants below 30°C (Ehleringer and Pearcy, 1983), which may permit C3 plants to maintain higher rates of CO_2_ fixation at low temperatures (Ehleringer, 1978). Under both high light and low temperature, the cold-adapted species *Muhlenbergia glomerata* (C4) and *Calamagrostis canadensis* (C3) showed a similar susceptibility to photoinhibition (Kubien and Sage, 2004) and this seems attributable to the ability of cold-adapted C4 species to activate the xanthophyll cycle (Kubien and Sage, 2004). Of note, although it is a reversible effect, such dynamic photoinhibition may reduce the photosynthetic performances of C4 in cold climates, thus reducing their competitiveness with C3 cold-adapted species and explaining their reduced distribution at high latitude and elevations.

What about the low relative abundance of C4 species and near-absence of C4 trees (Sage and Sultmanis, 2016)? Among other explanations (phenotypical, ecological, and evolutionary), the physiological hypothesis proposed C4 species to have a lower photosynthetic ability than C3 species in shade and/or fluctuating light conditions, such as in forest understoreys. In particular, it is conceivable that the main limiting factors consist of a lower quantum yield for CO_2_ (Ehleringer, 1978) and a reduced ability to use the fluctuating light efficiently (Kubásek et al., 2013). In fact, Kubásek et al. (2013) demonstrated that C3 and C4 species did not display photoinhibition when subjected to 950 μmol m^-2^s^-1^ light; however, only C4 species displayed photoinhibition (evidenced by the slight but significant reduction in *F*_v_/*F*_m_ ratio) when the same photon flux density was provided by simulated light fleck conditions over the whole experimental period. This was attributable to the photoinactivation of PSII and, in parallel, the activation of photoprotective mechanisms in C4 species. The photosynthetic metabolism in C4 species also responds less promptly to fluctuating light conditions because it requires more enzymatic steps than C3 to be light activated and to promote the metabolite gradient between MC and BSC (Sage and Pearcy, 2000). In addition, deactivation of Calvin cycle enzymes during low-light periods is faster in C4 than C3 species, causing faster reduction of CO_2_ quantum yield in C4 species in dynamic light conditions (Horton and Neufeld, 1998). Noteworthy, in C4 species under fluctuating light the CO_2_ quantum yield is additionally reduced by the increment of CO_2_ ‘leakiness’ in BSC, i.e., the proportion of CO_2_ fixed by PEPC in the mesophyll that leaks from BSC to the mesophyll without being re-fixed by Rubisco (Tazoe et al., 2008). These factors result in C4 species being inferior competitors to C3 species in conditions of shade or low-light conditions (flecked and dappled), and it can explain why C4 species are excluded from the ecological niche of the understory and why there are no C4 forests, an aspect which, besides biochemical limitations, seems principally attributable to a low phenotypic plasticity of C4 than C3 (Sage and McKown, 2006) species correlated with their shorter evolutionary history (Sage and Sultmanis, 2016).

## Actual Limits and Future Perspectives

As clearly evident by the two sections of this review, a gap exists between advances in chlorophyll fluorescence and the application of chlorophyll fluorescence parameters to improve knowledge on the mechanisms involved in photoprotection in C3 against C4 species. Surely, different morpho-anatomical and biochemical features in C4 compared with C3 species (and even different C4 metabolisms) further complicate this type of comparison. For this reason, we summarize below the main differences which actually limit the comparison between C3 and C4 metabolisms, propose the direction for future research toward this goal, and propose how chlorophyll fluorescence may assist in this research.

Firstly, while the ‘lack’ of photorespiration in C4 leaves increases the efficiency of Rubisco activity strongly, photorespiration is also essential for the protection of the photosynthetic apparatus of plants against photoinactivation under high irradiance (Heber et al., 1996). While photorespiration is assumed to be almost absent in C4 species under optimal conditions, it does appear to increase under conditions of limited CO_2_ availability, which can result from stresses which limit stomatal conductance (e.g., drought and salinity). For example, when wheat and maize plants were compared under 340 μbar CO_2_, photorespiration is fivefold higher in wheat as compared to maize. However, when the CO_2_ concentration was severely reduced (50 μbar intercellular CO_2_ concentration) photorespiration was substantially increased in both species (Dai et al., 1993). In contrast, studies have also reported that photorespiration did not contribute to reduced carbon gain in C4 species (Carmo-Silva et al., 2008; Lopes et al., 2011). In view of the higher involvement of the Mehler reaction in C4 species (compared to photorespiration) even at low O_2_ concentration (Laisk and Edwards, 1998), possible differences in the role of alternative sinks in C3 and C4 species should be considered. These factors show that there is a need to clarify the contribution of photorespiration as a possible alternative sink of electron transport rate when attempting to describe the intricate differences between C3 and C4 metabolisms under stomata-constraining stressors.

Secondly, gradients of PSI/PSII ratios differ substantially between C3 MC, C4 MC, and BSC, and even within C4 NAD-ME/NADP-ME metabolisms (Pfundel and Neubohn, 1999). This has serious consequences when attempting to describe the minute differences between the photochemical and non-photochemical processes in the thylakoid membranes of C3, C3-C4, and C4 species. Indeed, one should consider that values of *F*_v_/*F*_m_ are usually under-estimated because of the contribution of PSI to the overall signal of chlorophyll fluorescence (Pfündel, 1998), even at room temperature where PSI fluorescence was thought to be negligible (Krause and Weis, 1991). However, at room temperature it has been reported that the contribution of PSI fluorescence influenced both *F*_0_ and *F*_m_ in C3 and C4 species (Pfündel, 1998). This effect was consistent in C3, C3-C4, and C4 species, but in view of the relatively higher abundance of PSI in C4 species, *F*_0_ fluorescence accounted for 30% in C3 and 50% in C4 species (Pfündel, 1998). The abovementioned was consistent with the observation of a low chlorophyll fluorescence emission by PSII in NADP-ME species versus NAD-ME species (Takabayashi et al., 2005; Kirchhoff et al., 2013), the former having much lower levels of PSII in BSC. Therefore, we believe that for future research it is of crucial importance to deepen our understanding of influences to the photochemistry of PSI, for example by NIR-absorbance changes of PSI measured by the pulse amplitude modulation technique. In addition, it has been recently demonstrated that it is possible to differentiate between changes occurring to PSI, plastocyanin (PC) and ferredoxin (Fd) by a newly developed measuring system which combines the measure of chlorophyll fluorescence with the assessment of changes in NIR spectral regions (Klughammer and Schreiber, 2016). This allows the direct measurement of P700/PC, which has been demonstrated to strongly influence the rate of photosynthesis (Schöttler and Tóth, 2014), and provides for the first time direct measurement of Fd-dependent cyclic electron flow *in vivo* (Klughammer and Schreiber, 2016). The latter aspect is particularly relevant not only when comparing C3 versus C4 species, but also when comparing NAD-ME versus NADP-ME metabolisms, which are characterized by a different contribution between the two known pathways of cyclic electron flows around PSI: chloroplastic NAD(P)H dehydrogenase (NDH)-dependent flow and Fd-dependent flow (Takabayashi et al., 2005). In the past, the NDH-dependent pathway was measured by transient increase in chlorophyll fluorescence of PSII after turning off actinic illumination. However, due to the reduction of the plastoquinone pool by the reducing equivalents accumulated during actinic illumination (Takabayashi et al., 2005), it was impossible to assess the contribution of the Fd-dependent cyclic flow by chlorophyll fluorescence measurements.

Thirdly, when considering the different proportion between PSI/PSII ratios in C3 and C4 cells, it should be taken into account that the preferential distribution of PSII in stacked thylakoid regions (Dekker and Boekema, 2005) can significantly influence the LHCII mobility and the rate of repair of PSII when photodamaged, given that protein complexes in stacked regions (included LHCII and PSII components) have about a twofold lower mobility than unstacked regions of thylakoid membranes (Kirchhoff et al., 2013). In C3 species, photodamage to PSII occurs in stacked regions whereas the repairing mechanisms occur in unstacked regions, therefore requiring the mobility of PSII components (Tikkanen and Aro, 2012). This type of spatial separation between photodamage and repairing of PSII is not present in agranal BSC of C4 species, and it can explain the faster repairing of PSII in agranal BSC than MC in maize (Pokorska and Romanowska, 2007). As a consequence, despite NADP-ME metabolism is usually devoted to the production of ATP by cyclic electron transport and BSC are enriched in PSI and depleted of PSII, the faster capacity of the PSII repair process, connected to the PSII–PSI electron flux, should be considered when comparing C3 versus C4 species.

## Conclusion

The superiority of C4 over C3 photosynthesis under high light and elevated temperature is doubtless. Conversely, C4 species can be inferior competitors when subjected to other abiotic stressors, such as drought or cold, due to a higher susceptibility for photoinhibition. Assisted by chlorophyll fluorescence, future investigations should improve the knowledge on the different sensitivity of C3 and C4 to abiotic stressors; clarify as to whether photoinhibition occurs more frequently as a chronic or reversible process in C4; and deepen the understanding of light-induced reactions of C3 and C4 metabolisms with particular emphasis to the PSI photochemistry. This would give new perspectives and insights on the knowledge of C4 metabolism(s) under stress.

## Author Contributions

All authors listed have made a substantial, direct and intellectual contribution to the work, and approved it for publication.

## Conflict of Interest Statement

The authors declare that the research was conducted in the absence of any commercial or financial relationships that could be construed as a potential conflict of interest.
